# Thickness-Tunable Zoology of Magnetic Spin Textures Observed in Fe_5_GeTe_2_

**DOI:** 10.1021/acsnano.3c09602

**Published:** 2024-02-05

**Authors:** Ajesh K. Gopi, Abhay K. Srivastava, Ankit K. Sharma, Anirban Chakraborty, Souvik Das, Hakan Deniz, Arthur Ernst, Binoy K. Hazra, Holger L. Meyerheim, Stuart S.P. Parkin

**Affiliations:** † Max Planck Institute of Microstructure Physics, Weinberg 2, Halle (Saale) D-06120, Germany; ‡ Johannes Kepler University, Altenbergerstraβe 69, Linz 4040, Austria

**Keywords:** van der Waals materials, magnetic bubbles, skyrmions, bobbers, Lorentz transmission electron microscopy, Fe_5_GeTe_2_, fractal domains

## Abstract

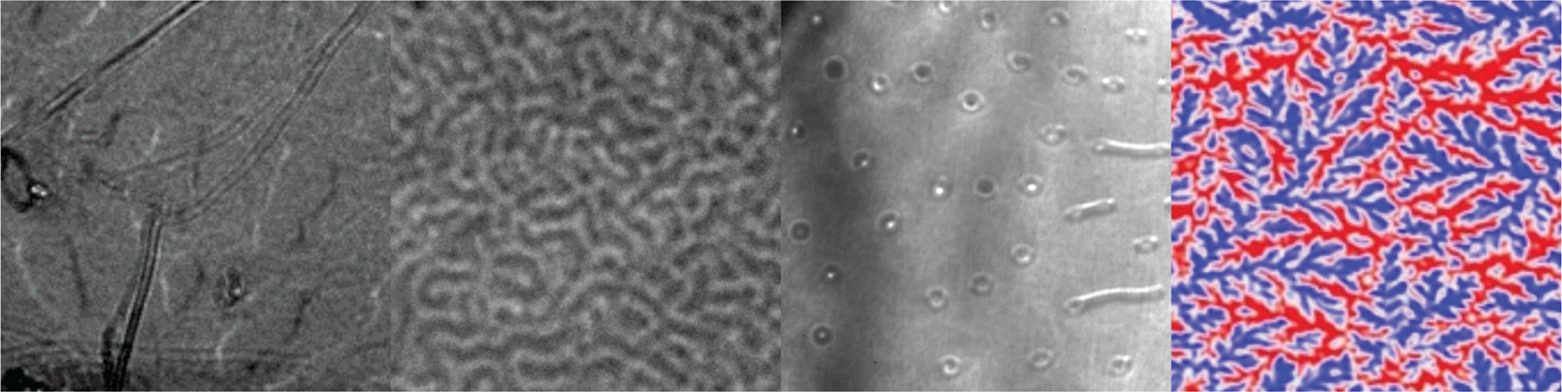

The family of two-dimensional (2D) van der Waals (vdW) materials provides a playground for tuning structural and magnetic interactions to create a wide variety of spin textures. Of particular interest is the ferromagnetic compound Fe_5_GeTe_2_ that we show displays a range of complex spin textures as well as complex crystal structures. Here, using a high-brailliance laboratory X-ray source, we show that the majority (1 × 1) Fe_5_GeTe_2_ (FGT5) phase exhibits a structure that was previously considered as being centrosymmetric but rather lacks inversion symmetry. In addition, FGT5 exhibits a minority phase that exhibits a long-range ordered (√3 × √3)-R30° superstructure. This superstructure is highly interesting in that it is innately 2D without any lattice periodicity perpendicular to the vdW layers, and furthermore, the superstructure is a result of ordered Te vacancies in one of the topmost layers of the FGT5 sheets rather than being a result of vertical Fe ordering as earlier suggested. We show, from direct real-space magnetic imaging, evidence for three distinct magnetic ground states in lamellae of FGT5 that are stabilized with increasing lamella thickness, namely, a multidomain state, a stripe phase, and an unusual fractal state. In the stripe phase we also observe unconventional type-I and type-II bubbles where the spin texture in the central region of the bubbles is nonuniform, unlike conventional bubbles. In addition, we find a bobber or a cocoon-like spin texture in thick (∼170 μm) FGT5 that emerges from the fractal state in the presence of a magnetic field. Among all the 2D vdW magnets we have thus demonstrated that FGT5 hosts perhaps the richest variety of magnetic phases that, thereby, make it a highly interesting platform for the subtle tuning of magnetic interactions.

## Introduction

The magnetic microstructure of a compound is a result of the interplay between several interactions that can give rise to a variety of spin textures, both achiral and chiral, and that includes domain walls, bubbles, merons, and a number of skyrmionic textures.^1−6^ For example, chiral skyrmionic spin textures in noncentrosymmetric magnets result from a competition between a Dzyaloshinskii-Moriya vector exchange interaction (DMI) and a ferromagnetic Heisenberg exchange interaction, whereas achiral skyrmionic bubbles in centrosymmetric magnets result from a competition between long-range dipole–dipole interactions and perpendicular magnetic anisotropy.^7^ Finding suitable materials where these interactions can be manipulated to give a desired spin texture is one of the prerequisites for technological applications.^8,9^ An example is the noncentrosymmetric Heusler compound Mn_1.4_Pt_0.9_Pd_0.1_Sn where the stabilization of elliptical skyrmions or antiskyrmions is controlled via a competition between the DMI and the dipole–dipole interaction.^10,11^ The family of two-dimensional (2D) magnetic van der Waals (vdW) materials^12−14^ provides a playground for the exploration of novel spin textures.^15^ Among these, the Fe_*x*_GeTe_2_ family is particularly interesting because it is metallic, exhibits a relatively high magnetic ordering temperature,^14,16,17^ and displays multiple spin textures depending on the Fe concentration.^18^ Recently, it was shown in the Fe_3_GeTe_2_ (FGT3) compound that there is an asymmetric distribution of Fe vacancies, together with vertical structural relaxations, that makes the structure noncentrosymmetric and, therefore, able to support Néel skyrmions.^19^ Another member of this family, Fe_5_GeTe_2_ (FGT5) with a higher Curie temperature (*T*_c_) of ∼300 K, has also been extensively investigated.^16,20−22^ Even though the compound has been shown to host magnetic stripe domains and skyrmionic bubbles,^23,24^ we show here that the zoology of spin textures is even richer than previously understood and can be controlled by a subtle tuning of the magnetic interactions via the thickness of the slab in which the textures are formed.

Using high-resolution X-ray diffraction (XRD) experiments, we find that FGT5 has a complex crystal structure composed of two distinct phases. The majority (1 × 1) structural phase lacks inversion symmetry, although the structure is very close to being centric as the atomic sites are shifted only slightly out of the atomic sites which are related by inversion symmetry. In addition, we find evidence for a minority phase embedded in this majority phase that exhibits a (√3 × √3)-R30° superstructure.^20^ Our analysis shows that this phase is characterized by an ordered array of Te vacancies in one of the two terminating Te layers of a FGT5 monosheet. We estimate the volume fraction of this minority phase to be quite small, in the 10^–2^ range relative to that of the majority phase. Using Lorentz Transmission Electron Microscopy (LTEM) and Magnetic Force Microscopy (MFM) we find that FGT5 flakes host a wide variety of spin textures that include a multidomain state, a stripe phase, and a fractal domain phase depending on the flake thickness. We also find evidence of unconventional type-I and type-II bubbles when a field is applied to the stripe phase and a bobber or a cocoon-like texture when a field is applied to the fractal phase. Furthermore, first-principles calculations based on the structural models that we have found provide unambiguous evidence that the strength of the DMI introduced by the small acentricity of the crystal structure is very small as compared to other skyrmion hosting bulk compounds. Thus, FGT5 provides a playground for devising complex spin textures due to, in particular, the weakness of the DMI interaction.

## Results and Discussion

The (1 × 1) structure of the majority phase of FGT5 was previously investigated, but contradictory results concerning the presence or absence of inversion symmetry were found in studies that used single crystals^25^ and powders.^17^ In the former, FGT5 was found to have an acentric space group (SGR) No. 160 (*R*3*m*), whereas the authors of the latter study concluded that their sample was best characterized by the centrosymmetric SGR No. 166 (*R*3̅*m*). This discrepancy was explained by different preparation procedures leading to different iron content in the samples.^17,20^

The structure of our samples was investigated using a platelike FGT5 single crystal (⌀ ≈ 2 mm, 100 μm thick) using a Ga-Jet X-ray source (λ = 1.341 Å) operated at 200 W power. Integrated reflection intensities were collected by a six-circle diffractometer^26^ using a focused beam provided by a Montel optics. Based on 54 symmetry-independent reflections (for more details see the Supporting Information) the structure parameters of the (1 × 1) phase were refined by least-squares refinement minimizing the unweighted residuum (*R*_U_) and the goodness-of-fit (GOF) parameter,^27^ the latter taking into account the ratio between the number of parameters and reflections. It was found that the measured intensities are fit best (*R*_U_ = 0.06, GOF = 1.23) with a structural model involving the acentric and polar SGR No. 160 (*R*3*m*), in which only the z-parameters of the atoms are allowed to vary. Refinement based on the centric SGR *R*3̅*m* (No. 166) leads to a much worse fit, quantified by GOF = 1.81. A schematic of the model is outlined in Figure S1 of Supporting Information. We find that the atomic positions do not deviate strongly from those expected for a centric cell (≈0.008 to 0.026 lattice units at most). Therefore, we denote the structure as “weakly” acentric. We emphasize that the structural relaxations of the atoms make the individual FGT5 sheets themselves acentric and polar (reduction of the point group symmetry from 3̅2/*m* to 3*m*), *i*.*e*., the two terminating Te layers become inequivalent, which is important in the context of the (√3 × √3)-R30° superstructure which is discussed in the following.

**Figure 1:**
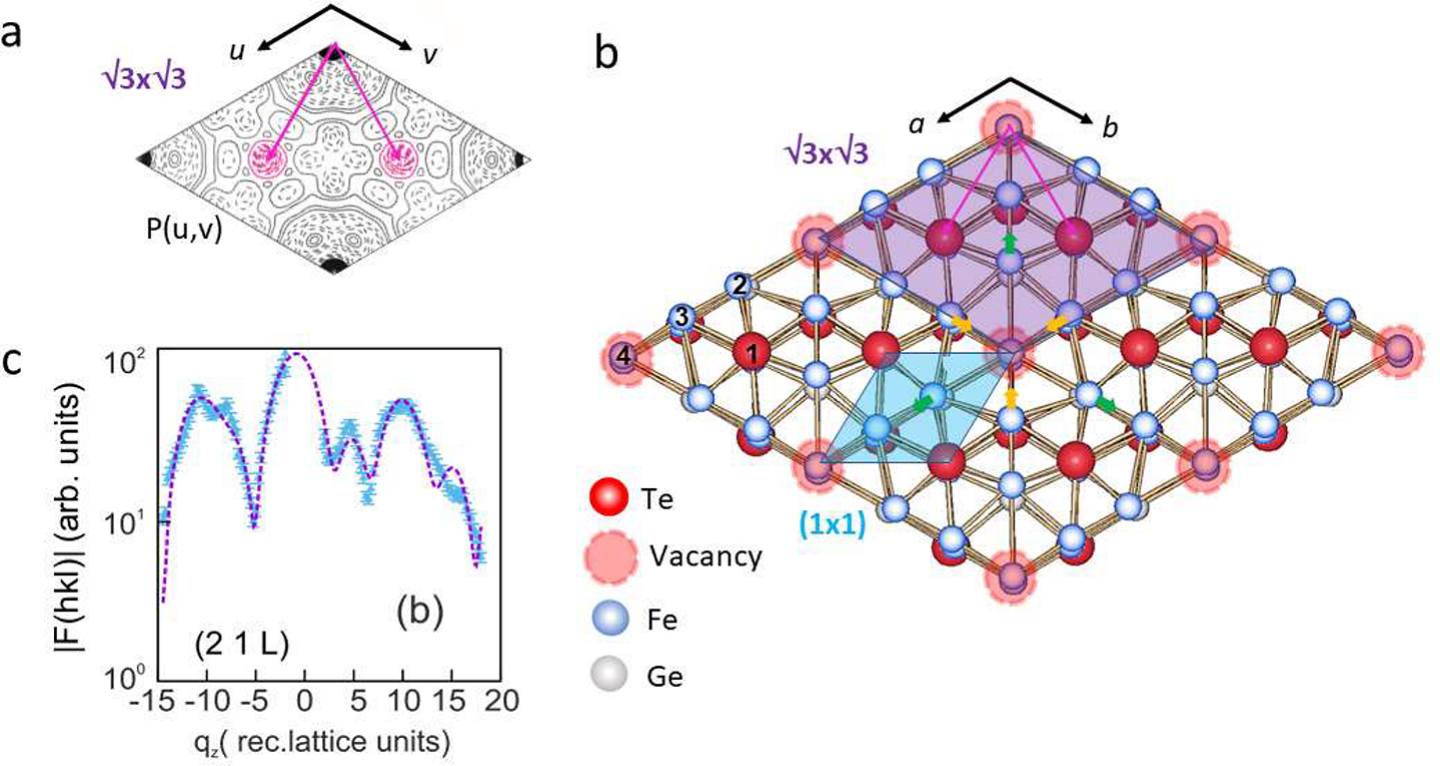
X-ray structural analysis of the (√3 × √3)-R30° superstructure. (a) z-projected Patterson function *P*(*u*,*v*) showing the interatomic correlations within the unit cell (uc). Apart from the trivial positive maximum at the origin, only one nontrivial symmetry-independent negative maximum (red dashed lines, arrows) is observed, directly indicating a Te vacancy. This is the main characteristic of the superstructure. (b) Structural model showing four (√3 × √3)-R30° unit cells close to top view: one unit cell corresponds to the area highlighted in purple. One (1 × 1) uc is rather shown by the blue shading. Pink arrows indicate the correlation vector related to *P*(*u*,*v*) in (a). Atoms are numbered according to Table 1 of the Supporting Information. The sequence of the atoms along [0001] corresponds to that of the bulk. (c) Small yellow and green arrows indicate relaxations of Fe atoms next to the Te vacancy. Variation of the |F(21L)| structure factor magnitude in log-scale along *q*_*z*_. The continuous distribution reflects the 2D character of the structure.

For the (√3 × √3)-R30° superstructure 233 symmetry-independent reflection intensities, *I*(*hkl*), were collected, of which 21 are of the type (*hk*0). The structure factor magnitudes (|*F*(*hkl*)|) were derived from the *I*(*hkl*) by applying instrumental factors.^28^ Using the |*F*(*hk*0)|, the z-projected Patterson-function *P*(*u*,*v*) of the superstructure was calculated according to , where *u* and *v* are the z-projected components of the interatomic vectors of the atoms within the unit cell. *P*(*u*,*v*) is shown in Figure 1(a) and is compared with the structural model in Figure 1(b) where the violet area represents the unit cell. Red, blue, and gray balls represent Te, Fe, and Ge atoms, respectively. *P*(*u*,*v*) provides direct and model-free information concerning the interatomic vectors within the unit cell.^26^

Our *P*(*u*,*v*) exhibits only two prominent maxima within the asymmetric unit (two of which form the entire unit cell), namely, (i) the trivial positive (solid lines) maximum at (*u*,*v*) = (0,0) corresponding to the self-correlation of all atoms and (ii) an intense negative maximum (red dashed lines) at (*u*,*v*) = (^2^/_3_,^1^/_3_) which directly indicates the presence of a vacancy. Other features in *P*(*u*,*v*) are comparatively weak in amplitude and can be viewed as resulting from truncation errors in the Fourier-summation due to the limited number of reflections. Thus, from the negative correlation related to the interatomic vector (^2^/_3_, ^1^/_3_) one can directly conclude that a Te vacancy is the main structural characteristic of the (√3 × √3)-R30° superstructure.

The structural model was subsequently refined using only one of the FGT5 sheets having a Te vacancy at the origin of the unit cell as the starting model. The superstructure is purely 2D since the intensity along *q*_*z*_ is continuously distributed as shown by the representative example of the (21L) reflections in Figure 1(c). The maxima can be viewed as Bragg peaks resulting from the positive interference of the X-rays scattered from the atomic layers within the FGT5 sheet. The best fit to the observed |*F*(*hkl*)| is represented by the dashed line, which well-reproduces those observed. For all 233 reflections, the overall fit quality is given by *R*_U_ = 0.12. The final structural model is schematically outlined in Figure 1(b) which shows four (√3 × √3) unit cells viewed almost along the surface normal. Atoms are labeled by numbers according to Table S1 of the Supporting Information, which provides a complete list of the atomic positions in comparison with that of the (1 × 1) unit cell. The latter is indicated by the blue area in Figure 1(b).

The superstructure is mainly characterized by an ordered array of Te vacancies in one of the terminating layers of an individual FGT5 sheet, which corresponds to the Wyckoff site (1a) (0,0,*z*) in SGR *P*31*m*. There is one vacancy per (√3 × √3) unit cell; i.e., one-third of the Te atoms are missing. In Figure 1(b) the vacancy site is emphasized by the red open spheres. Neighboring Fe atoms relax toward and away from the vacancy which in Figure 1(b) is emphasized by colored arrows. The atomic shifts out of the bulk (1 × 1) positions are typically a few hundredths of the unit cell size, *i*.*e*., approximately 0.05–0.25 Å. Local structural disorder of several Fe and Ge atoms is also observed and considered by anisotropic Debye–Waller factors, but disorder is not the main characteristic of the superstructure. As discussed in more detail in the Supporting Information, the superstructure is present throughout the volume of the crystal and is not confined to the crystal’s surface. Based on the analysis of the Te concentration in the majority (1 × 1) structure (see Supporting Information) we estimate the volume fraction of the superstructure to lie in the 10^–2^ range. We tentatively suggest that it is formed only at the surface of the microcrystals from which the bulk sample is composed. This considerably diminishes the contribution of the superstructure to any emergent DMI that may arise due to the strong inversion asymmetry resulting from the fact that a vacancy is present in only one of the two terminating Te layers.

The magnetic microstructure was investigated by both LTEM and MFM using FGT5 flakes that were prepared using two different techniques: (i) Focused Ion Beam (FIB) milling and (ii) a standard scotch tape technique with heat-assisted exfoliation^29^ (see Methods). For the LTEM measurements, exfoliation was carried out directly onto a 50 nm thick Si_3_N_4_ membrane grid so that multiple flakes of different thicknesses were available. For these measurements, the samples were cooled from room temperature (above *T*_c_) to the measurement temperature of 100 K in a zero magnetic field. This temperature was chosen so that there is sufficient magnetic contrast (i.e., a big enough magnetization). The defocus value was 1.5 mm unless otherwise specified.

**Figure 2:**
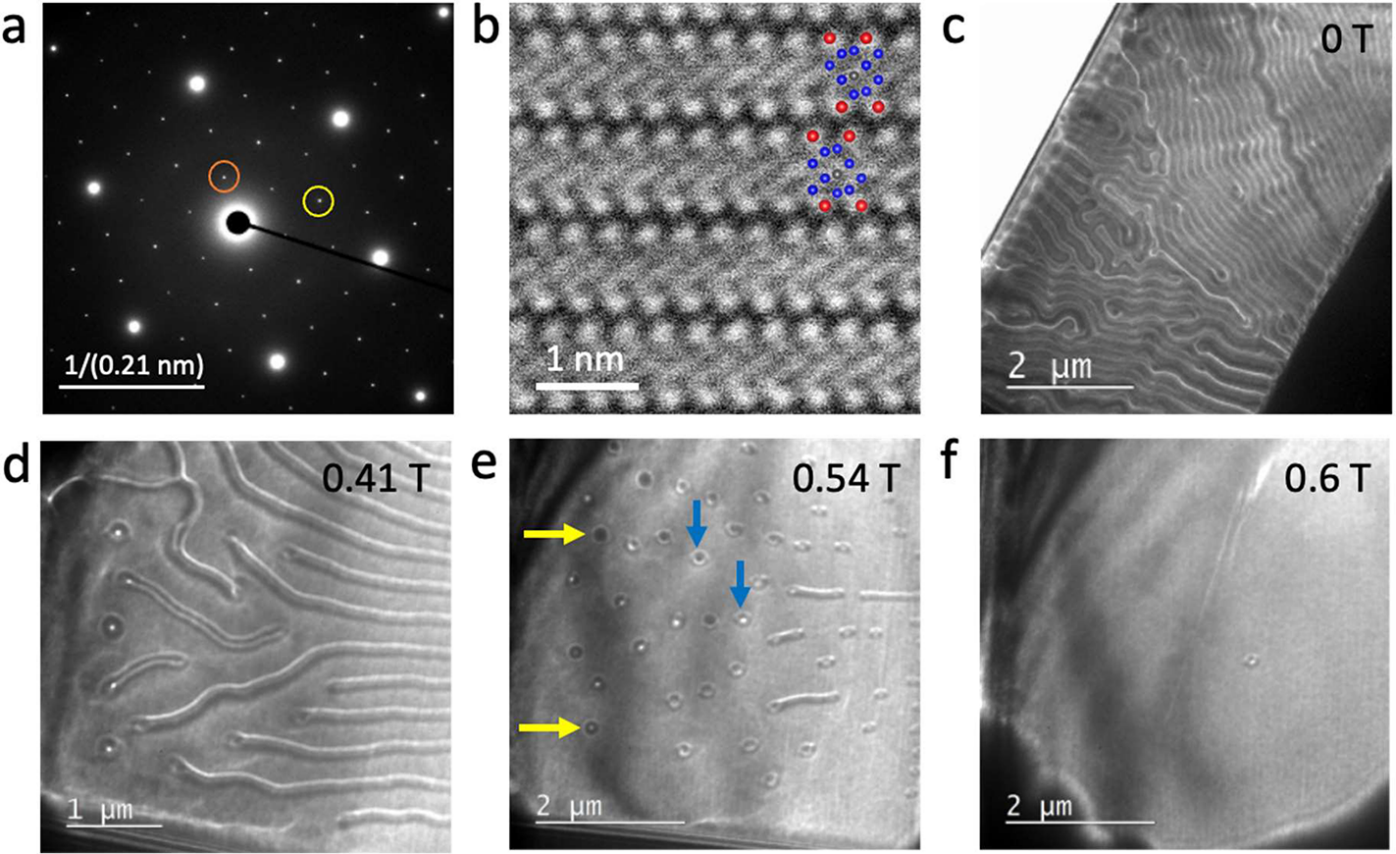
(a) Electron diffraction pattern obtained along the [001] direction showing sharp (1 × 1) (marked with yellow circle) and (√3 × √3) (marked with orange circle) superstructure reflections. (b) Experimental HAADF-STEM image showing the atomic arrangements along the [100] direction. Approximate fitting of the atoms is shown for comparison. Atoms are represented by colored spheres: red (Te), blue (Fe), and gray (Ge). LTEM images of an FGT5 flake, ∼200 nm thick, recorded at 100 K and with a 1.5 mm under-focus in an applied out of plane field of (c) 0 T, (d) 0.41 T, (e) 0.54 T, and (f) 0.6 T. Yellow and blue colored arrows in (e) point to unconventional type-I and type-II bubbles, respectively.

We first discuss the FIB prepared lamella. Figure 2(a) shows an electron diffraction pattern obtained from a lamella with a [0001] orientation. This lamella was ∼200 nm thick, with a slight thickness gradient from one edge to another. Sharp reflections related to both the (1 × 1) majority structure and the (√3 × √3)-R30° superstructure are observed, indicating a highly long-range structural order. Figure 2(b) shows an experimental HAADF-STEM image showing the atomic arrangements along the [101̅0] direction. The inset shows the corresponding placement of the atoms. Figure 2(c) shows an LTEM overview of the entire lamella, where magnetic stripe domains are observed. The width of the stripes increases from the top-right to the bottom-left corner of the image, presumably a consequence of the thickness gradient across the lamella. Subsequently, an out-of-plane magnetic field was applied. With increasing field, the stripe domains expand, followed by the formation of various types of magnetic bubbles, as shown in Figure 2(d–f).

The bubbles observed in Figure 2(e) appear, at first sight, to be similar to type-I and type-II bubbles that have been observed previously in several inversion-symmetric compounds.^30−34^ However, a careful inspection of the contrast in the LTEM images reveals that these are distinct. In a type-I bubble, a cylindrical boundary exists that separates the center of the bubble from the outside.^35^ In the center, the moments point along the opposite direction relative to those at the periphery of the bubble (Supporting Information Figure S4). Within the boundary, the spins rotate in either a clockwise or a counterclockwise direction so that the bubble can have a topological charge of either +1 or −1. The bubbles are stabilized by the competition between the perpendicular magnetic anisotropy and dipolar interactions.^35^ The type-I bubble has a very similar spin texture as a Bloch skyrmion.^36^ The only difference is that there is no DMI that governs the chirality or the sense of rotation of the moments. Therefore, both chiralities can exist that are winding either clockwise or counterclockwise within the outer rim of the bubble. When a small in-plane field is applied, type-I bubbles transform into a slightly different texture, a type-II bubble, in which two Bloch lines are formed in the bubble boundary (see Supporting Information Figure S4).^30,37^ Therefore, the moments do not complete a full rotation, and the topological charge becomes zero. Simulated spin textures and LTEM contrasts of type-I and type-II bubbles are shown in Figure S4 of the Supporting Information. The LTEM contrast of these bubbles does not match those that we observe in FGT5 so that we, therefore, conclude that these bubbles are distinct from previously observed bubbles. In the following, we describe them as “unconventional” bubbles.

**Figure 3:**
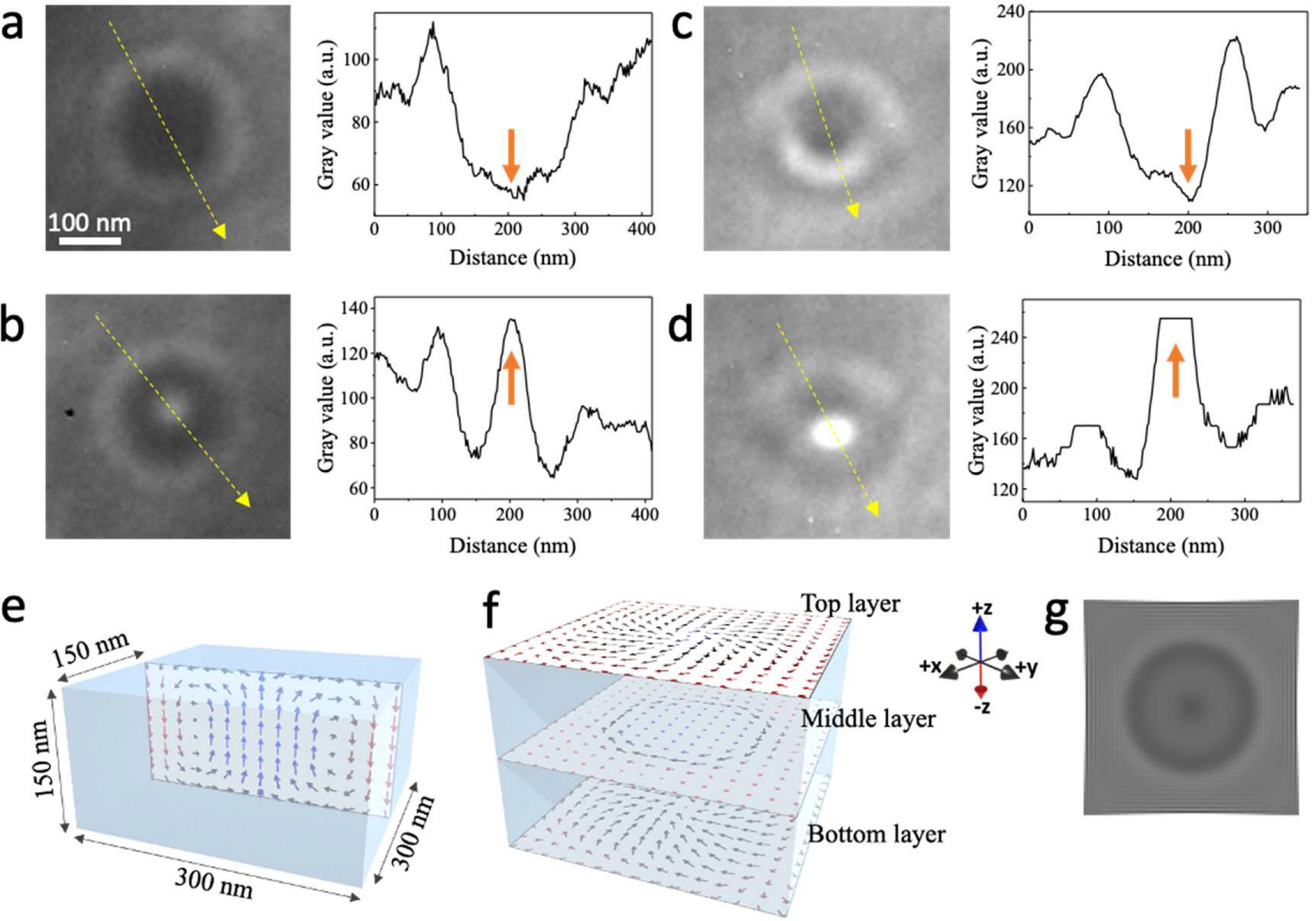
Magnified LTEM image and corresponding line profiles of (a, b) unconventional type-I and (c, d) unconventional type-II bubbles indicated by the yellow and blue arrows in the LTEM images shown in Figure 2(d). Line profiles were measured along the direction of the arrows in the LTEM images. Orange arrows highlight the position of the bright or dark contrast at the center of bubbles. The scale bar (100 nm) for all LTEM images is the same and is shown in (a). Simulated spin texture showing (e) a cross-section and (f) the magnetization components in the top, middle, and bottom layers of an unconventional type-I bubble and the corresponding simulated LTEM contrast (g) showing an additional dark contrast in the center. Color of the arrows in (e, f) represents the direction of the moments: blue (+*z*), black (*x*-*y* plane), and red (−*z*) as shown in the inset of (f). Image (f) is scaled in the *z*-direction for a better view of the middle and bottom layers.

Figure 3(a–d) shows magnified LTEM images and line profiles of the bubbles marked in Figure 2(e). Figure 3(a,b) shows unconventional type-I bubbles where the moments in their boundary complete a full rotation. However, the central region of these bubbles has an additional bright or dark contrast, as compared to conventional type-I bubbles. Unconventional type-II bubbles are shown in Figure 3(c,d). For these bubbles, the outer boundary has the same texture as a conventional type-II bubble but the central region again shows an additional brighter or darker contrast. The line profiles of both the type-I and type-II bubbles show a clear dip or elevation of the contrast in their center, respectively, again distinguishing them from their conventional counterparts.

To better understand the spin textures of these unconventional bubbles, we performed micromagnetic simulations, which were then used to simulate the LTEM contrast and compared with our experimental observations. For the simulations we chose a simulation volume with an area of 300 × 300 nm^2^ by 150 nm thickness and material parameters that are provided in the Methods section. We find unconventional bubbles that are not uniform throughout the thickness of the simulated region but rather have a cylindrical structure in the interior with a Bloch-like boundary, but in the surface regions the moments are mostly twisted in-plane with the moments in the central regions pointing upward, opposite to the moments outside the object’s boundaries, as shown in Figure 3(e,f). This resembles a Meron-like structure. The spin twisting as a function of thickness results from a competition between anisotropy and dipole–dipole interactions in uniaxial magnets with what is referred to as a quality factor , where *K*_u_ is the uniaxial magnetic anisotropy and *M*_s_ is the saturation magnetization.^38,39^ This spin twisting gives the bubbles a complex 3D structure that results in an additional contrast in the center of the LTEM image of the bubble. Figure 3(g) shows the simulated LTEM image of this unconventional type-I bubble that is characterized by an additional dark contrast in the center, in agreement with our experimental observations (Figure 3a). Similarly, for the unconventional type-II bubbles, Supporting Information Figure S6(a,b) shows the simulated cross-section and in-plane component of the top, middle, and bottom layers. The spin twisting in the near surface region can be seen. Supporting Information Figure S6(c) shows the simulated LTEM contrast, which matches well with our experimental images (Figure 3d).

We note that refs (40 and 41) have reported real space observations of Néel-type skyrmions using LTEM in a [0001] oriented flake of F_5–*x*_GeTe_2_. Ref (40) reports that Néel skyrmions are observed only for lower thicknesses (∼61 nm) which, it was argued, were due to a DMI interaction at the surfaces of their flake due to a (√3 × √3) superstructure caused by vertical atomic Fe ordering. However, our structural analysis provides unambiguous evidence that the superstructure is a result of ordered Te vacancies instead of Fe ordering. Ref (41) shows Néel skyrmions and merons in different regions of the flake having out-of-plane and in-plane anisotropy, respectively. These regions clearly show the inhomogeneous nature of the crystal itself, and therefore, their results cannot directly be compared with our results.

Next, we explored the magnetic microstructure as a function of thickness. LTEM images collected using much thinner FGT5 flakes than those discussed above are shown in Supporting Information Figure S7. The images were taken from a ∼25 nm thick flake prepared by using mechanical exfoliation rather than by FIB, which was employed to prepare the thicker samples discussed so far. At 100 K and without any applied field, large domains are observed that are random in shape and size, which is at variance with the stripe phase discussed above for much thicker lamellae. Upon application of a perpendicular magnetic field, these domains are gradually transformed into the field polarized state, and no bubble phase was observed during this process (see Supporting Information Figure S7). This indicates that the phase observed here is inherently different from the stripe phase. LTEM images of another flake containing regions with three different thicknesses (56, 71, and 116 nm) are shown in Supporting Information Figure S8. These images show a stripe phase in zero field for all three thicknesses, but the width of the stripes increases with increasing thickness. Note that a similar result was observed for a FIB prepared lamella, as shown in Figure 2(c). An increase in the width of the stripe phase as a function of increasing lamella thickness has been attributed to the presence of long-range dipole–dipole interactions in Mn_1.4_PtSn: both the period of the helical phase as well as the skyrmion size were found to increase with lamella thickness.^7^ A similar dependence of the size of Néel-like skyrmion on lamella thickness was subsequently observed in two other compounds PtMnGa^4^ and Fe_3_GeTe_2_^19^ (another member of the 2D magnetic materials family).

**Figure 4:**
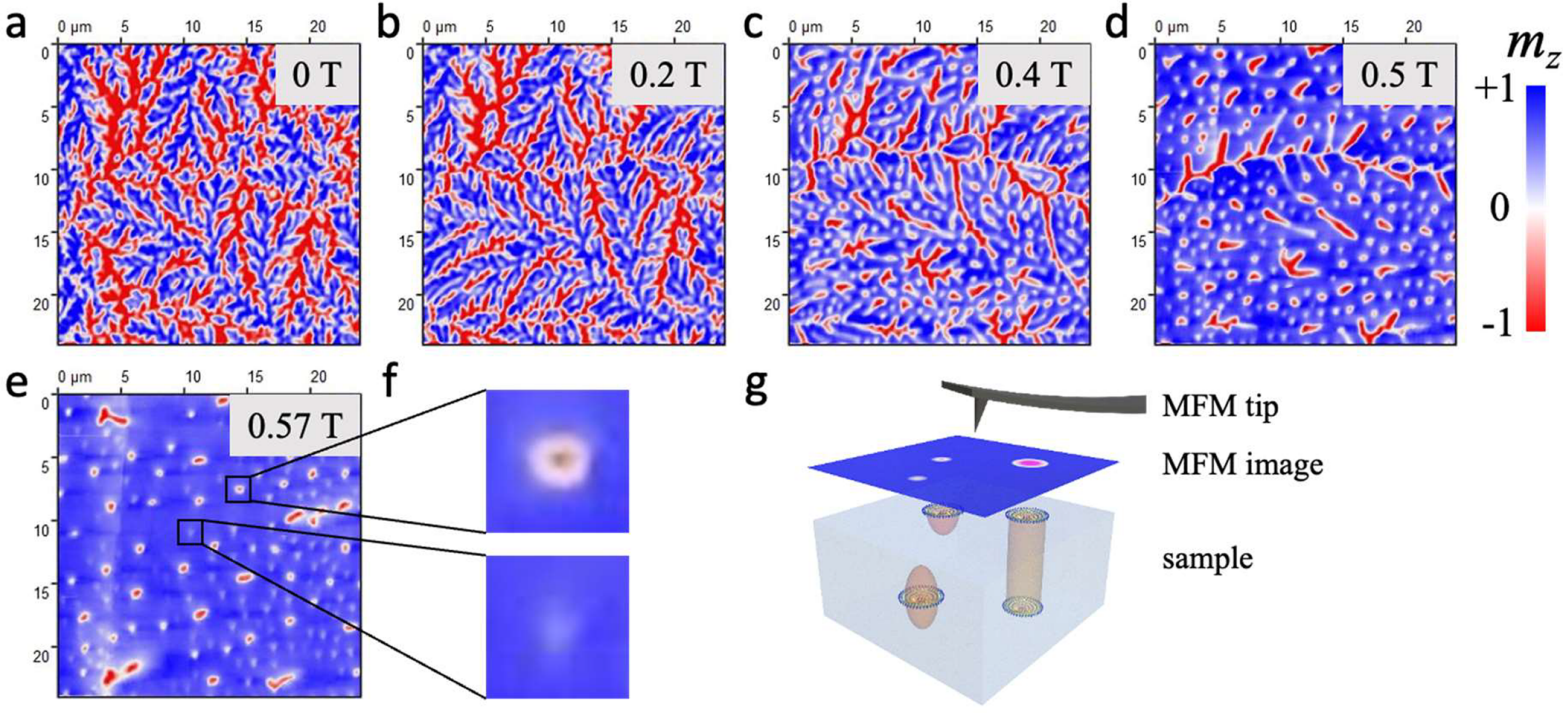
(a–e) MFM images of a bulk crystal with thickness ∼170 μm recorded at 100 K in the presence of an external field. Numbers in the upper right corner of each image show the corresponding field strength. (f) Magnified images of two selected regions (2 μm × 2 μm) in (e) showing two distinct magnetic contrasts. (g) Schematic representation showing the sample hosting different magnetic textures that result in different magnetic contrasts in MFM measurements.

As LTEM is limited to thin samples, we employed MFM measurements to investigate the presence of magnetic texture in bulk (thickness ∼170 μm) crystals along the [0001] direction. MFM image contrast results from the interaction of the MFM tip magnetic moment with the stray field generated from the sample surface. For the detection of the out-of-plane component, we used a tip magnetized in the direction perpendicular to the sample surface. As outlined above, the sample was cooled to 100 K in the absence of an external field. MFM images recorded for the bulk crystal are shown in Figure 4. The colors in the MFM images represent the magnetization components along different directions: blue (up), red (down), and white (in-plane).

In zero external field, an interesting fractal-like magnetic texture is observed, as shown in Figure 4(a). On increasing out-of-plane field, the fractal texture starts to evolve into skyrmionic bubble-shaped objects randomly distributed throughout the sample, as shown in Figure 4(b–e). Interestingly, two distinct magnetic contrasts are observed at higher field. Figure 4(f) shows the magnified view of two distinct skyrmionic textures observed in Figure 4(e). One appears as the two oppositely magnetized regions (blue and red) separated by a white circular boundary, whereas the other appears as a much weaker white contrast. The appearance of these two distinct contrasts at the same field and sample thickness shows that they result from two distinct magnetic textures.

We find that similar MFM images have been reported in distinct thin-film multilayer structures.^42,43^ Since MFM can only sense the stray field at the surface, micromagnetic simulations were used to help to understand the 3-dimensional nature of the observed spin textures. It was shown that two distinct features in the MFM result from two spin textures that can be stabilized simultaneously. One is a tube-shaped skyrmion that goes through all the layers, and the other is a bobber-like structure at the surface or a cocoon-like structure buried within the layers, as illustrated in Figure 4(g). We find great similarity between this prior work and the observed MFM contrast in our bulk FGT5 even though the thickness here is ∼170 μm, as compared to the prior thin-film multilayer systems with a thickness <200 nm. Carrying out micromagnetic simulations for our very thick samples is computationally very expensive.

We note that similar fractal domains have been observed in the hard ferromagnet Nd_2_Fe_14_B.^44,45^ We also note that the thickness dependence for FGT5 and Co-doped FGT5 has been reported before,^46,47^ but none of these studies provide a complete picture of the zoology of magnetic textures that exist as a function of thickness. Our LTEM and MFM observations reveal three different thickness regimes, where three distinct magnetic ground states are observed.

Our experimental findings are supported by first-principles theoretical calculations in which the strength of the DMI, the *D*/*A* ratio, was calculated where *A* is the spin stiffness and *D* is the projection of the spiralization matrix *D* onto the direction of the rotation vector. The parameters *A* and *D* were obtained from the generalized Heisenberg exchange tensor,^48^ which was calculated using the generalized magnetic force theorem,^49−51^ as implemented within multiple scattering theory.^52^ The diagonal elements of the tensor represent the nearest neighbor (NN) Heisenberg exchange parameter, *J*_*ij*_, while a superposition of the off-diagonal tensor elements corresponds to the NN DMI exchange constant, *D*_*ij*_. The estimated *D*/*A* ratio for the (√3 × √3)-R30° superstructure was found to be too small to allow for bulk DMI stabilized skyrmion formation (*D*/*A* = 0.147 nm^–1^). This is because although the crystalline structure is acentric, atomic relaxations around the Te vacancy are small and do not lead to a large *D*/*A* ratio. Without the Te vacancies, the *D*/*A* ratio is even smaller by a factor of 2 (*D*/*A* = 0.073 nm^–1^). For the (1 × 1) FGT5 phase, the *D*/*A* ratio was found to be 0.022 nm^–1^, which is even smaller by another factor of 3 than that calculated for the (√3 × √3)-R30° phase. The calculations fully support our experimental finding that only bubbles rather than skyrmions are observed, which is related to the absence of a significant DMI interaction.

## Conclusion

In conclusion, we find that the FGT5 structure lacks inversion symmetry in both the (1 × 1) phase and (√3 × √3)-R30° superstructure phase. While for the (1 × 1) phase we find only slight vertical relaxations out of the atomic positions related by inversion symmetry, the (√3 × √3)-R30° superstructure phase results from an ordered array of Te vacancies in one of the two terminating layers of the FGT5 sheet. The volume fraction of the latter is estimated to be of the order of 1%. Therefore, we suggest that the acentric (√3 × √3)-R30° phase plays no important role as a possible contributor to the DMI, while in the case of the (1 × 1)-phase the DMI is weak compared to other skyrmion hosting compounds. LTEM and MFM measurements carried out on lamellae of different thicknesses reveal three distinct magnetic ground states that are stable for different thicknesses: (i) domain phase, (ii) stripe phase, and (iii) fractal phase. We also observe unconventional type-I and type-II bubbles where the spin texture is twisted through the thickness of the lamella. Furthermore, we find the presence of a bobber-like feature at the surface or a cocoon-like structure buried within the layers of very thick FGT crystals. Our results show that FGT5 hosts a zoology of magnetic textures that can be tuned by the flake thickness. A combination of metallicity, room temperature *T*_c_, thickness dependence, and presence of a variety of magnetic phases makes FGT5 a promising material for future spintronic applications.

## Methods

### FIB

For TEM and LTEM measurements, lamellae were prepared using a Ga+ focused ion beam system TESCAN GAIA 3 operating in the range of 0.5 to 30 keV ion beam energy. Standard lift-out procedures were employed to extract the lamella from bigger flakes using a nano manipulator and transferred to a grid. The lamella was then polished to the desired shape and thickness. In a final step, the lamella was polished with an ion beam at low energy (3–5 keV) to reduce any surface damage caused during preparation.

### Flakes Preparation

Our crystals were purchased from the company HQ Graphene. These crystals were prepared by using a CVT method. Details of the fabrication process are given in ref [^17^]. We find that the basic properties of our FGT5 samples above ∼200 nm in thickness (exfoliated or FIBed from single crystals) are stable in an ambient environment for long periods of weeks to months. On the other hand, the properties of thin flakes with thicknesses below ∼15 nm may be altered within a few days. Nevertheless, short exposures (∼15 min) do not much change the properties of the flakes, whether thick or thin. All the sample preparation including exfoliation and transfer was carried out in a nitrogen glovebox with less than 1 ppm of oxygen and less than 2 ppm of water content. The samples were stored in a controlled environment to minimize exposure to oxygen until measurements were performed. To determine the thickness, Atomic Force Microscopy (AFM) scans were performed on the samples after the magnetic measurements.

### TEM/LTEM

FEI-TITAN 80-300 and JEOL F200 electron microscopes operating at 300 and 200 keV, respectively, were employed for the TEM and LTEM measurements. A double tilt holder with a liquid nitrogen cooling option was used for measurements at temperatures ranging from 100 to 300 K.

### MFM

MFM measurements were performed in an attoAFM I variable-temperature MFM microscope (attoLIQUID2000) equipped with a vector superconducting magnet. A magnetic tip from Nanosensors (model SSS-MFMR) with a tip radius of ∼20 nm was used for all measurements. MFM images were recorded in constant height mode. First, the topography of the sample was acquired in tapping mode after correcting the tilt and misalignment of the sample. Then, the MFM tip was lifted by 30–50 nm from above the sample during a second scan to measure the magnetic signal in noncontact mode. The phase shift of the cantilever, caused by the magnetic interactions, was detected by using the phase modulation method.

### XRD

The detailed crystal structure of the FGT5 crystal was analyzed by employing a custom-made six-circle diffractometer and a Gallium-Jet X-ray source (λ = 1.3414 Å) operated at 70 keV and 200 W power. The beam was focused onto the sample under grazing incidence (μ = 1°) by Montel optics providing a highly collimated beam of 100 μm height and 2 mm width. Integrated reflection intensities were collected using transverse phi-scans of the sample and a two-dimensional pixel detector. Data collection along the superlattice rods was carried out by line scans along the *q*_*z*_-direction in reciprocal space.

### Micromagnetic Simulations

The Object Oriented Micromagnetic Framework (OOMMF) code was used to simulate the magnetic texture of the unconventional bubbles. For the simulations we chose a sample size of 300 × 300 × 150 nm^3^ with a cell size of 3 × 3 × 3 nm^3^. Material parameters were used as follows: Exchange constant (*A*) is 1.1 × 10^–11^ J m^–1^, out-of-plane uniaxial anisotropy (*K*_u_) is 50 kJ m^–3^, saturation magnetization (*M*_s_) is 700 kA m^–1^, damping constant (α) 0.5. We did not include any DMI in the simulations. For both unconventional bubbles, we started with an initial configuration of either a type-I or a type-II bubble and allowed the simulations to run until a stable state was achieved.

### First-Principles Calculations

First-principles calculations were performed using a self-consistent fully relativistic Green function method^53^ within the generalized gradient approximation for the exchange-correlation potential.^54^ Disorder effects were taken into account within a coherent potential approximation as implemented in a multiple scattering theory.^55^ The generalized Heisenberg exchange tensor was estimated using the magnetic force theorem^49^ adopted for the relativistic case.^50,51^

## Supporting Information Available

XRD analysis, LTEM image simulations of conventional and unconventional bubbles and LTEM images of several flakes with different thicknesses (PDF)
