# Supplementary material for: Thickness-Tunable Zoology of Magnetic Spin Textures Observed in Fe5GeTe2
Source: ACS Nano. 2024 Feb 5;18(7):5335–43. doi: 10.1021/acsnano.3c09602 (PMC10883052; doi:10.1021/acsnano.3c09602)
Supplement: Supplementary file 1 — nn3c09602_si_001.pdf [file nn3c09602_si_001.pdf]

Supporting Information

Thickness tunable zoology of magnetic spin textures observed in Fe_5GeTe_2

Ajesh K. Gopi[1†], *Abhay K. Srivastava*[1†], *Ankit K. Sharma*[1,3†], *Anirban Chakraborty*[1], *Souvik Das*[1], *Hakan Deniz*[1], *Arthur Ernst*[2], *Binoy K. Hazra*[1], *Holger L. Meyerheim*[1], *Stuart S.P. Parkin*[1*]

[1]Max Planck Institute of Microstructure Physics, Weinberg 2, Halle (Saale) D-06120, Germany

[2]Johannes Kepler University, Altenbergerstraβe 69, Linz 4040, Austria

[*]Email: stuart.parkin@mpi-halle.mpg.de

X-ray diffraction analysis of the FGT5 structure

1. Analysis of the bulk (1x1) structure

The bulk (1x1) structure of Fe_5GeTe_2 (FGT5) was previously investigated in several studies, namely by Stahl *et. al.* [1] and later by a powder diffraction analysis of May *et al.*,[2] but contradictory results concerning the presence or absence of inversion symmetry were reported. While in Ref.[1] the crystal was found to belong to the acentric space group (SGR) #160 ($R3m$), the latter authors came to the conclusion that the structure of their sample is centrosymmetric [SGR #166 ($R\overline{3}m$)]. This discrepancy might be explained by different preparation procedures leading to different iron content in their samples.

Our experiments were carried out on a plate like crystal using a Ga-jet X-ray source (λ=1.341 Å) and a six-circle diffractometer, as described in Ref.[3] In total 104 reflections were collected reducing to 54 by symmetry equivalence. The structural refinement was carried out by least squares fit of the calculated structure factor magnitudes (|Fcalc|) to the observed ones |(Fobs)|). For the best fit an unweighted residual (Ru) of 0.06 and a goodness of fit (GOF) of 1.23 was obtained.[4] The structural model is shown in Figure S1.

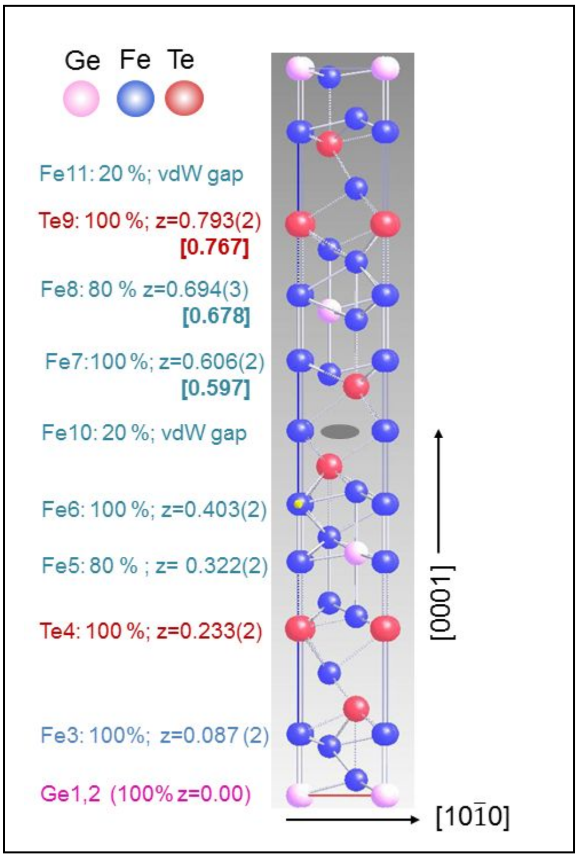

Figure S1: Model of the FGT5-(1x1) structure: Schematic view of one-unit cell of FGT5. Symmetry independent atoms within the unit cell (space group *R*3*m*) which are located at (x,y,z)=(0,0,z) are listed on the left together with their z-positions and their respective site occupancy factors in percent. Uncertainties are given in brackets referring to the last digit. Bold numbers in rectangular brackets for atoms #7, 8, 9 refer to calculated z-coordinates of these atoms in the case of a centrosymmetric structure.

The atoms are labelled by the sequence of their positions along the *c*-axis. The most important result is that a two-fold rotation axis [see symbol in Figure S1] which would relate the upper and the lower half of the unit cell is missing. This is indicative for the absence of inversion symmetry. We note however that this asymmetry is only "weak" as the related atoms are only slightly shifted in the range between 0.009 and 0.026 lattice units (see values in brackets for atoms #7,8 and 9) out of their inversion symmetric positions. All symmetry independent atoms reside in the 3a Wyckoff site at (00z) of space group (SGR) $R3m$ (#160), involving only one free parameter (z). The atomic positions for the atoms as well as the structural disorder closely resemble those published previously.[1]

In addition, we find some fraction of Fe atoms within the van der Waals (vdW) gaps, similar to the case of the recently investigated Fe_3GeTe_2 (FGT3) crystal[5] where about 20-30% of Fe is located in the vdW gaps. These sites are labelled by "Fe10" and "Fe11". This extra fraction of Fe within the vdW gaps is counterbalanced by the presence of Fe defects (≈20%) at sites labelled by "Fe5" and "Fe8". Therefore, the FGT5 crystal is close to the ideal Fe_5GeTe_2 stoichiometry. Using this model, the |Fobs| can be fitted very well which is represented by Figure S2(a) showing the plot of |Fcalc| versus |Fobs|. The diagonal line represents the ideal condition |Fcalc| = |Fobs|.

We have further addressed the structural details such as the site occupancies (SOF) of the different sites by Fe and Te atoms together with their uncertainties. To this end we have calculated contour plots in which R_U is plotted versus site occupancies at different positions as shown in Figure S2(b)-(d)

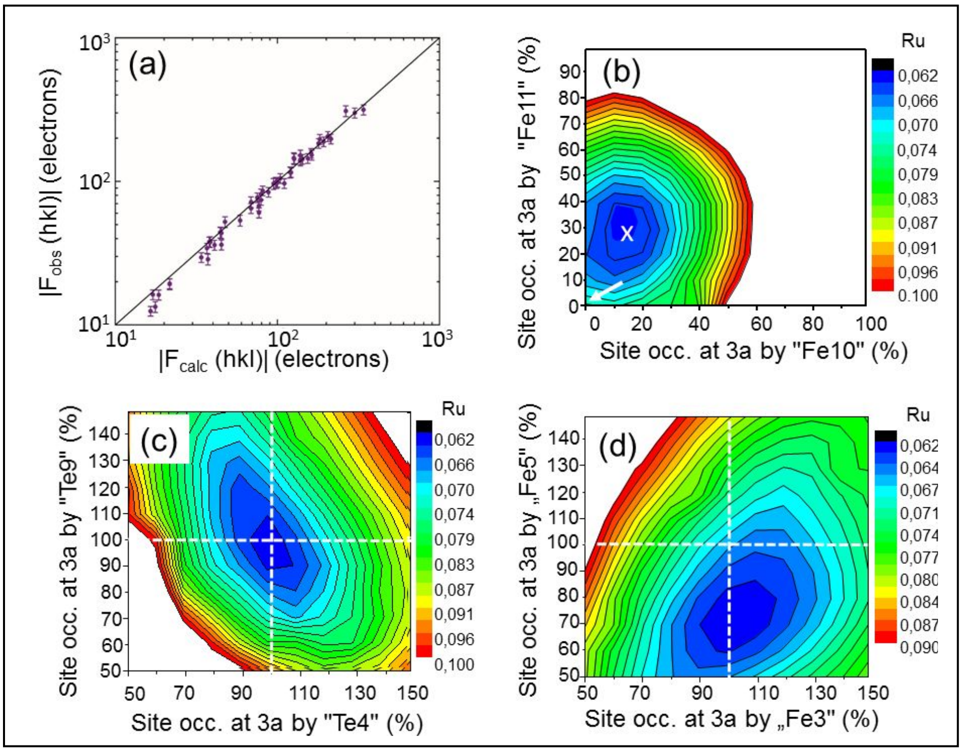

Figure S2: Detailed analysis of the FGT5 (1x1) structure: (a) Plot of |Fobs| versus |Fcalc| for all 54 symmetry independent reflections based on the best fit model discussed in Figure S1. The data points closely follow the diagonal line representing the ideal condition |Fobs|=|Fcalc|. The unweighted residuum (R_U) is equal to 0.061. (b)-(d): Contour plots of R_U versus site occupancy (SOF) of Fe and Te atoms for different sites [see also Figure S1(a)]. The white dashed lines in (c) and (d) represent the 100% level corresponding to full site occupancy. (b) shows the SOF of Fe atoms in the vicinity of the vdW gaps.

At first, Figure S2(b) clearly indicates the SOF of 20-30% of Fe at sites labelled by "Fe10" and "Fe11", respectively [see Figure S1]. The improvement of R_U by taking into account this amount of Fe at sites near the vdW sites significantly improves the fit quality from about 7.4 % (see the arrow for the case that both sites have SOF=0) to 6.1%. Similarly, we find defects (i.e. SOF less than 100% for the regular Fe positions) at "Fe5", but not for the "Fe3" site. This is somewhat at variance with the results of Ref.[1] (see Figure S2(d)). Finally, we have studied the Te SOF's, which are close to 100% for both sites [see Figure S2(c)]. This provides an estimate for the volume fraction of the (√3×√3)-R30° superstructure within the sample. Since

in the (√3×√3)-R30° superstructure one third of the "Te9" or the "Te4" atoms are missing, therefore, the SOF of these sites should be reduced to 0.66 if the superstructure would be present throughout the volume of the sample. A SOF of 0.66 is clearly not observed, but the refined SOF values are close to 100% (uncertainty of the SOF determination lies in the 5-10 percentage point regime). As a consequence, the upper limit of the volume fraction of the superstructure is estimated to lie in the 10^{-2} range. This quantification suggests a structural model, in which the sample is composed of microcrystals ("mosaic crystals") where only the outer surfaces of a mosaic crystals exhibit the (√3×√3)-R30° superstructure. As the Te defects and the structural relaxations involve a significant breaking of the inversion symmetry of the (1x1) bulk structure one might argue that the superstructure contributes to the Dzaloshinskii-Moriya Interaction (DMI). However, we suggest that its low volume fraction considerably diminishes its overall contribution to the DMI.

2. Analysis of the (√3×√3)-R30° superstructure

The structural model is discussed in the main text. The full data set and the calculated |F(hkl)| using the model shown in Figure 1 of the main text are shown in Figure S3.

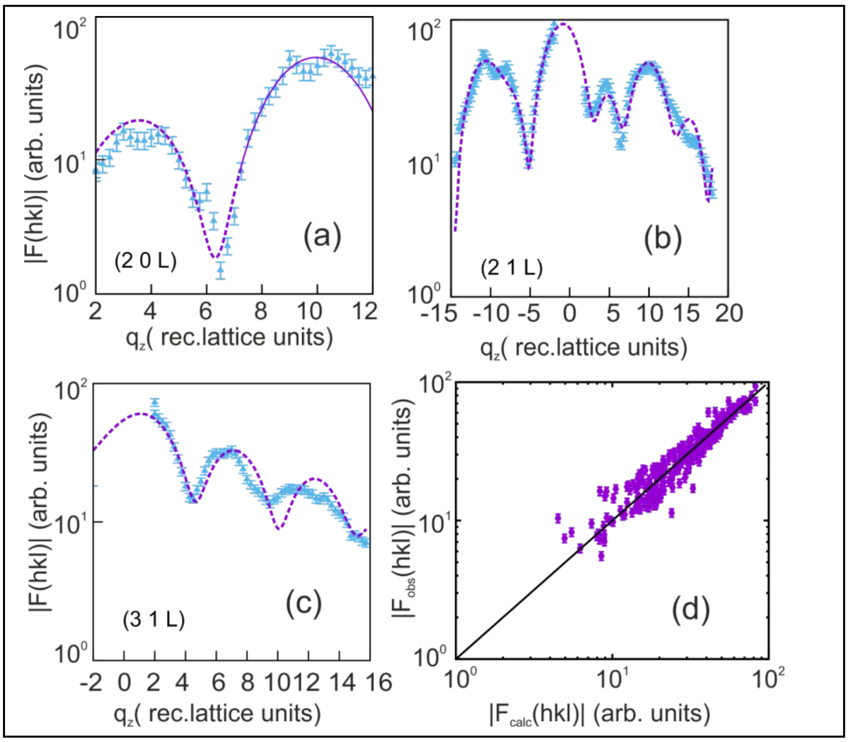

In

Figure S3: Analysis of the (√3×√3)-R30° superstructure. Data and best fit: (a)-(c): Experimental (blue symbols) and calculated (lines) structure factor magnitudes (|F(hkl)|) along several superlattice rods on a log scale. The calculation is based on the structural model shown in Figure 1 of the main text. (d): Plot of |F(calc)| versus |F(obs)| for all 233 reflections.

The full data set including the in-plane reflections of type (hk0) plus several superlattice rods consists of 233 independent reflections. The intensity is continuously distributed along q_z as the periodicity along the [0001] direction is missing. Three superlattice rods are shown in Figure S3(a-c), while the fit for the whole data set is represented in the |Fcalc| versus |Fobs| plot shown in figure S3(d). The best fit to the rods is represented by the dashed lines, which fairly well reproduces the steep intensity variation along q_z. The overall fit quality is expressed by the unweighted residuum (R_U),[4] which is equal to 0.12 and can be considered as very good.

Table S1 summarizes the refined positions together with the atomic disorder expressed by the Debye parameter (B) which is given by: $B=8\pi^2\times U$, where U is the mean squared displacement of the atoms from its equilibrium position.[6] These are quite large and anisotropic ($B^{11}\neq B^{33}$) for some atoms, (e.g. for #6 (Ge) and #3,4 (Fe) in agreement with previous study.[1] Atom labels refer to the model shown in Figure 1(a) of the main text.

Nr	Species<br>(Wyckoff site)	Θ	X	y	z	B(Å^2)
1	Te (2b)	1.00	2/3*<br>2/3	1/3*<br>1/3	0.273*<br>0.279	13±1

2	Fe (3c)	1.00	0.31±0.01<br>1/3	0.0*<br>0.0	0.266±0.001<br>0.237	4±1
3	Fe (3c)	1.00	0.60±0.01<br>2/3	0.0*<br>0.0	0.239±0.002<br>0.229	19±2
4	Fe (1a)	0.85	0.0*<br>0.0	0.0*<br>0.0	0.212±0.003<br>0.190	13±2
5	Fe(2b)	0.85	2/3*<br>2/3	1/3*<br>1/3	0.212±0.003<br>0.190	13±2
6	Fe (3c)	1.00	0.29±0.01<br>1/3	0.0*<br>0.0	0.173±0.001<br>0.142	2±1
7	Ge (3c)	1.00	0.63±0.01<br>2/3	0.0*<br>0.0	0.139±0.001<br>0.151&0.173	B^{11}=2±1<br>B^{33}=22±4
8	Fe (1a)	1.00	0.0*<br>0.0	0.0*<br>0.0	0.109±0.001<br>0.103	7±1
9	Fe(2b)	1.00	2/3*<br>2/3	1/3*<br>1/3	0.109±0.001<br>0.103	7±1
10	Te (3c)	1.00	0.32±0.01<br>1/3	0.0*<br>0.0	0.082±0.001<br>0.051	1.8*

Table S1: Coordinates of the atoms in the ($\sqrt{3}\times\sqrt{3}$)-R30° superstructure: Refined positions and Debye parameters (B) of the atoms within the supercell. Coordinates in red letters refer to the (1x1) bulk-position of the atoms.[1] Lattice parameters are *a*=*b*=6.99 Å, *c*=29.19 Å, where *c* corresponds to the FGT5 bulk lattice parameter which was taken as a reference. Numbers refer to Figure 1 in the main text, labels in parentheses refer to the Wyckoff position in the two-dimensional space group *P*31*m*. Parameters marked with asterisks were kept fixed.

LTEM image simulation

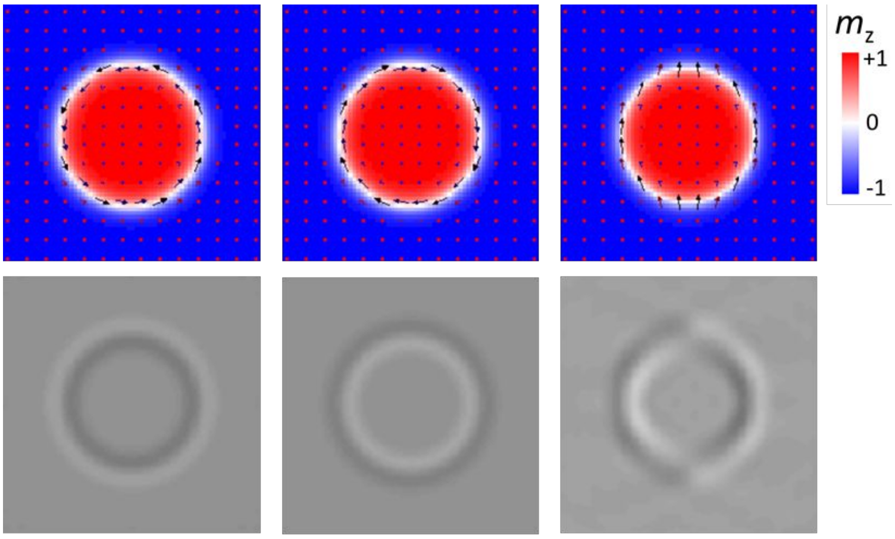

Type-I (CCW) Type-I (CW) Type II

Figure S4: Simulated spin texture and corresponding simulated LTEM image of type-I (with counter-clockwise (CCW) or clockwise (CW) rotating domain boundary) and type-II bubbles. Micromagnetic simulation software OOMMF[7] was used to generate the magnetization configurations shown here. This configuration was used with MALTS[8] to generate simulated LTEM contrast.

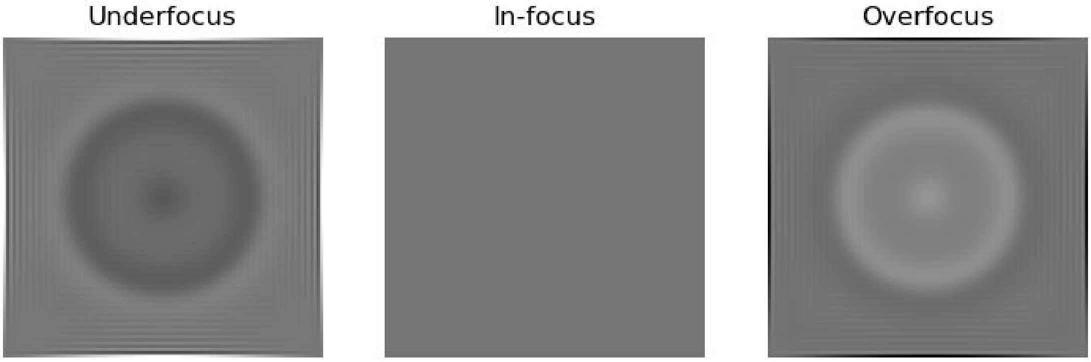

Figure S5: Simulated LTEM contrast of the unconventional type-I bubble showing an additional bright/dark region in the center of the bubble.

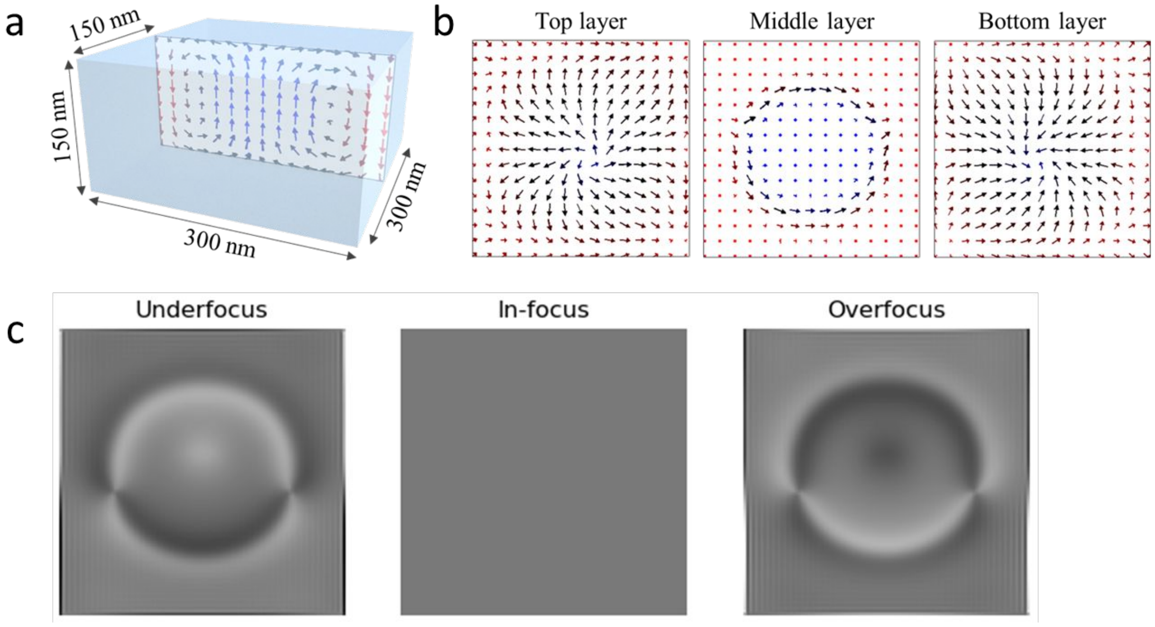

Figure S6: Micromagnetic simulation showing the (a) cross-section and (b) magnetization component in the top, middle and bottom layer of an unconventional type-II bubble. (c). Corresponding simulated LTEM contrast showing an additional bright/dark region in the center of the bubble. We used PyLorentz code to simulate the LTEM contrast of the unconventional bubbles.[9]

Effect of field on domain phase

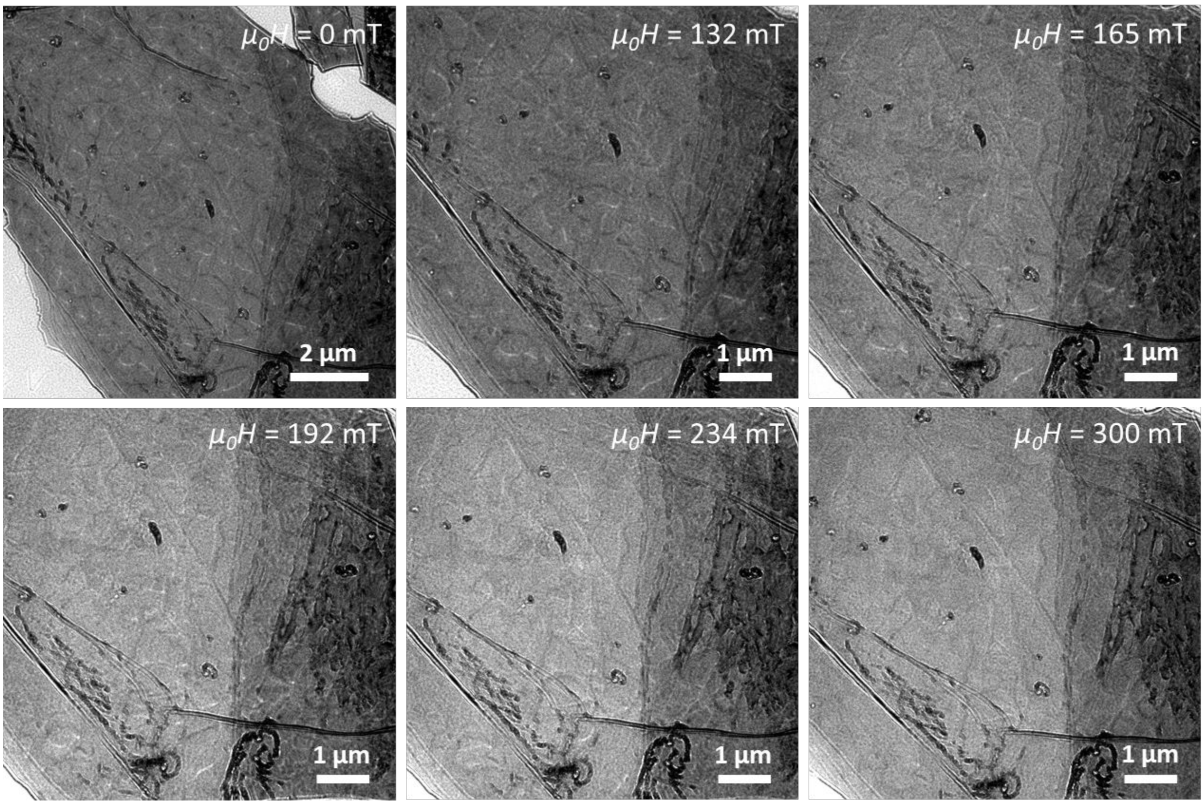

Figure S7: A sequence of LTEM images was recorded at different fields for a flake of thickness ~25 nm. Large domains of random shape can be seen in the image. These domains can be recognized by bright and dark contrast appearing at the domain boundary. With increasing field, the domain size becomes smaller and smaller and finally transforms into a field-polarized state. These images were recorded at 100 K and 1.5 mm defocus.

Flakes with different thicknesses

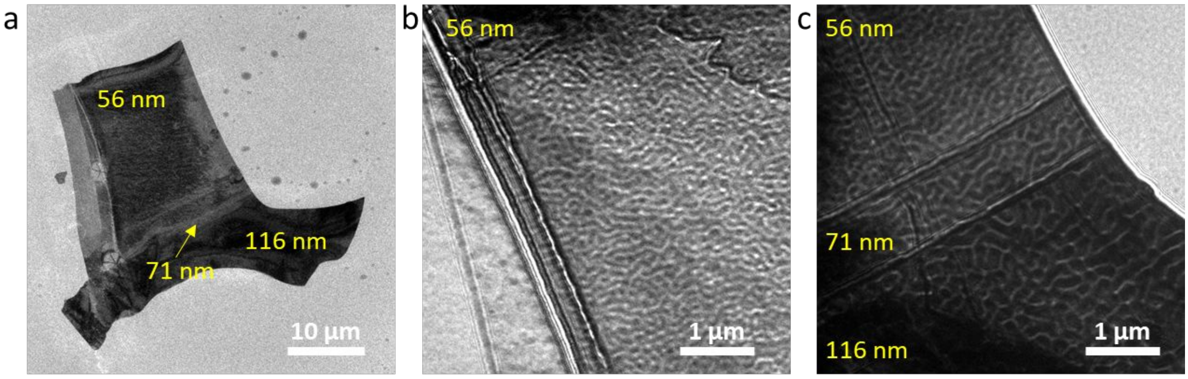

Figure S8: LTEM images of a flake characterized by different thicknesses across its area. (a) Overview of the flake, (b) magnified view of the thin (56nm) region and (c) magnified view showing all three different thicknesses. Numbers written in each image indicates the thickness of the corresponding region. It can be seen that with increasing flake thickness, the width of the stripe domains increases. These images were recorded at 100 K, 1.5 mm defocus and no external field.

References:

1. Stahl, J.; Shlaen, E.; Johrendt, D., The van der Waals ferromagnets Fe5–δGeTe2 and Fe5–δ–xNixGeTe2–crystal structure, stacking faults, and magnetic properties. *Z. Anorg. Allg. Chem.* **2018,** *644* (24), 1923-1929.
2. May, A. F.; Ovchinnikov, D.; Zheng, Q.; Hermann, R.; Calder, S.; Huang, B.; Fei, Z.; Liu, Y.; Xu, X.; McGuire, M. A., Ferromagnetism near room temperature in the cleavable van der Waals crystal Fe5GeTe2. *ACS nano* **2019,** *13* (4), 4436-4442.
3. Moritz, W.; Van Hove, M. A., *Surface Structure Determination by LEED and X-rays*. Cambridge University Press: 2022.
4. Woolfson, M. M.; Woolfson, M. M., *An introduction to X-ray crystallography*. Cambridge University Press: 1997.
5. Chakraborty, A.; Srivastava, A. K.; Sharma, A. K.; Gopi, A. K.; Mohseni, K.; Ernst, A.; Deniz, H.; Hazra, B. K.; Das, S.; Sessi, P., Magnetic Skyrmions in a Thickness Tunable 2D Ferromagnet from a Defect Driven Dzyaloshinskii–Moriya Interaction. *Adv. Mater.* **2022,** *34* (11), 2108637.
6. Kuhs, W., Generalized atomic displacements in crystallographic structure analysis. *Acta Crystallogr. Sect. A: Found. Crystallogr.* **1992,** *48* (2), 80-98.
7. Donahue, M. J.; Donahue, M., *OOMMF user's guide, version 1.0*. US Department of Commerce, National Institute of Standards and Technology: 1999.
8. Walton, S. K.; Zeissler, K.; Branford, W. R.; Felton, S., MALTS: a tool to simulate Lorentz transmission electron microscopy from micromagnetic simulations. *IEEE transactions on magnetics* **2013,** *49* (8), 4795-4800.
9. McCray, A. R.; Cote, T.; Li, Y.; Petford-Long, A. K.; Phatak, C., Understanding complex magnetic spin textures with simulation-assisted Lorentz transmission electron microscopy. *Phys. Rev. Appl.* **2021,** *15* (4), 044025.
